# European Society for Organ Transplantation (ESOT) Consensus Statement on the Use of Non-invasive Biomarkers for Cardiothoracic Transplant Rejection Surveillance

**DOI:** 10.3389/ti.2024.12445

**Published:** 2024-06-11

**Authors:** Andriana Nikolova, Sean Agbor-Enoh, Saskia Bos, Marisa Crespo-Leiro, Stephan Ensminger, Marta Jimenez-Blanco, Annamaria Minervini, Michael Perch, Javier Segovia, Robin Vos, Kiran Khush, Luciano Potena

**Affiliations:** ^1^ Smidt Heart Institute, Cedars-Sinai Medical Center, Los Angeles, CA, United States; ^2^ Genomic Research Alliance for Transplantation (GRAfT) and Laboratory of Applied Precision Omics, National Heart, Lung, and Blood Institute (NHLBI), NIH, Bethesda, MD, United States; ^3^ Lung Transplantation, Department of Medicine, Johns Hopkins Hospital, Baltimore, MD, United States; ^4^ Newcastle University Translational and Clinical Research Institute, Newcastle uponTyne, United Kingdom; ^5^ Institute of Transplantation, Newcastle Upon Tyne Hospitals NHS Trust, Newcastle uponTyne, United Kingdom; ^6^ Cardiology Department, Complexo Hospitalario Universitario A Coruna (CHUAC), Instituto de Investigación Biomédica A Coruña (INIBIC), Universitade de Coruna (UDC), Centro de Investigación Biomédica en Red—Enfermedades Cardiovasculares/Network Biomedical Research Center—Cardiovascular Diseases (CIBERCV), La Coruna, Spain; ^7^ Klinik für Herz- und Thorakale Gefäßchirurgie, Universitäres Herzzentrum Lübeck, Lübeck, Germany; ^8^ Cardiology Department, University Hospital Ramón y Cajal (Madrid), Centro de Investigación Biomedica en Red—Enfermedades Cardiovasculares (CIBERCV), Madrid, Spain; ^9^ Heart Failure and Transplant Unit, IRCCS Azienda Ospedaliero-Universitaria di Bologna, Bologna, Italy; ^10^ Department of Cardiology, Section for Lung Transplantation, Righospitalet, Copenhagen, Denmark; ^11^ Department of Clinical Medicine, University of Copenhagen, Copenhagen, Denmark; ^12^ Cardiology Department, Puerta de Hierro Majadahonda University Hospital, Universidad Autónoma de Madrid, Instituto de Investigación Sanitaria Puerta de Hierro-Segovia de Arana/Puerta de Hierro Health Research Institute—Segovia de Arana (IDIPHISA), Centro de Investigación Biomédica en Red—Enfermedades Cardiovasculares/Network Biomedical Research Center—Cardiovascular Diseases (CIBERCV), Madrid, Spain; ^13^ Department of Respiratory Diseases, UZ Leuven, and Lung Transplant Unit, Department of Chronic Diseases and Metabolism, Laboratory of Respiratory Diseases and Thoracic Surgery (BREATHE), KU Leuven, Leuven, Belgium; ^14^ Division of Cardiovascular Medicine, Stanford University School of Medicine, Palo Alto, CA, United States

**Keywords:** heart transplantation, lung transplant, biomarker, rejection, guidelines

## Abstract

While allograft rejection (AR) continues to threaten the success of cardiothoracic transplantation, lack of accurate and repeatable surveillance tools to diagnose AR is a major unmet need in the clinical management of cardiothoracic transplant recipients. Endomyocardial biopsy (EMB) and transbronchial biopsy (TBBx) have been the cornerstone of rejection monitoring since the field’s incipience, but both suffer from significant limitations, including poor concordance of biopsy interpretation among pathologists. In recent years, novel molecular tools for AR monitoring have emerged and their performance characteristics have been evaluated in multiple studies. An international working group convened by ESOT has reviewed the existing literature and provides a series of recommendations to guide the use of these biomarkers in clinical practice. While acknowledging some caveats, the group recognized that Gene-expression profiling and donor-derived cell-free DNA (dd-cfDNA) may be used to rule out rejection in heart transplant recipients, but they are not recommended for cardiac allograft vasculopathy screening. Other traditional biomarkers (NT-proBNP, BNP or troponin) do not have sufficient evidence to support their use to diagnose AR. Regarding lung transplant, dd-cfDNA could be used to rule out clinical rejection and infection, but its use to monitor treatment response is not recommended.

## Introduction

Despite major advances in the management of immunosuppression, allograft rejection (AR) continues to threaten the success of cardiothoracic transplantation. AR can lead to acute immune-mediated graft dysfunction, as well as chronic multifactorial graft-specific diseases, such as cardiac allograft vasculopathy (CAV) and chronic lung allograft dysfunction (CLAD), both ultimately leading to graft failure and death.

Lack of accurate and repeatable surveillance tools to diagnose AR is a major unmet need in the clinical management of cardiothoracic transplant recipients. Endomyocardial biopsy (EMB) and transbronchial biopsy (TBBx) have been the cornerstone of rejection monitoring since the field’s incipience. Long considered the “gold standard,” both suffer from significant limitations, including sampling error, high cost, potential complications, and patient discomfort. Moreover, prior studies have shown poor overall concordance of biopsy interpretation among pathologists [1].

The vast majority of TBBx and EMB performed during surveillance do not show signs of clinically meaningful AR, hence highlighting the need for reliable non-invasive biomarkers to screen for AR and to reduce the frequency of invasive procedures [2, 3].

A multitude of biomarkers for rejection diagnosis have been developed over the past few decades and are at different stages of commercial development and clinical validation. Given these advances in the field, a working group was convened by the European Society of Organ Transplantation (ESOT) that included healthcare professionals from across Europe and North America with expertise in the field. The panel has reviewed the existing literature for the degree of evidence supporting the use of these assays in clinical practice in order to provide clinical practice recommendations for the clinical use of biomarkers in cardiothoracic transplant rejection surveillance, and to highlight knowledge gaps that need to be fulfilled by future research.

In this context, the working group has chosen to focus the discussion and recommendations mainly on emerging biomarkers assayed by molecular biology techniques (i.e., the gene expression profiling (GEP) test AlloMap and donor-derived cell-free DNA [dd-cfDNA] assays), given their commercial availability as diagnostic tests and the initial use in clinical practice (Table1). In addition, two cardiac biomarkers [troponin and B-type natriuretic peptides (BNP)] which have been re-examined in recent studies as to their utility for rejection surveillance in heart transplantation, are discussed.

**TABLE 1:** Practical considerations in the use of the currently commercially available GEP and dd-cfDNA assays.

Assay	Type of rejection monitoring and diagnostic thresholds	Validation studies	Timing of initiation	Suitable patient populations to be applied to	Caveats
**Allomap (CareDx)**—peripheral blood mononuclear cells–based 11-gene expression panel (ITGAM, FLT3 and IL1R2 are steroid responsive)	Validated only for ACR monitoring (not for AMR) with score range 0–40 >99% NPV for ACR with the following diagnostic thresholds ≥30 for patients 2–6 months post HT and ≥34 after 6 months post HT	Validated in 2 randomized (IMAGE and eIMAGE) clinical trials and large prospective observational cohort studies (both US and European based- OAR and CARGO II) as non-inferior to EMB for rejection surveillance	Per the IMAGE and eIMAGE studies, eligible patients include those ≥55 days post HT and on <20 mg of daily prednisone dose, and up to 5 years post HT.	-HT recipients >15 years of age with normal graft function (LVEF≥50%) and asymptomatic -No history of AMR≥1 or treated ACR Grade ≥2R -Absence of DSAs -On corticosteroid dose <20 mg/day -Have not received hematopoietic growth factors or blood transfusions during the previous 30 days -Are not pregnant -No history of severe CAV -Absence of CMV infection (both asymptomatic viremia or CMV disease)	-Different test thresholds can be chosen to maximize either sensitivity or specificity per the clinicians’ needs -The GEP test has not been validated against intragraft gene expression -Affected by other factors leading to immune activation (steroid dose, infections, leukopenias, etc.) -In the USA, it is processed in centralized laboratories -Adoption of the test in Europe is limited by cost considerations and establishing laboratory infrastructure for testing
**Allosure (CareDx), Prospera (Natera)** **Allonext (Eurofins)**—dd-cfDNA assays measuring the fraction of donor derived cfDNA compared to the recipient’s cfDNA	Can be applied to both AMR and ACR monitoring >97% NPV for AR with the following diagnostic thresholds Allosure ≥0.20% and Prospera ≥0.15% Allonext >0.15	No randomized controlled trials have tested the non-inferiority of dd-cfDNA-based vs. EMB-based monitoring for AR (upcoming DETECT trial (NCT05081739 will address that – uses Prospera) Validated in 3 large prospective cohort studies conducted in North America (upcoming FreeDNA-CAR (NCT04973943) will compare cdDNA vs. EMB based surveillance in centers in Spain)	In the GRAfT study (uses research-grade assay), patients were enrolled at ≥28 days post HT, in D-OAR (uses Allosure)—>55 days post HT and in DEDUCE (uses Prospera)—≥28 days post HT. Most centers implement dd-cfDNA testing starting 1–3 months post HT Threshold values for HT recipients monitoring >2 years post HT are undefined	-Single HT organ recipients only (not tested in multi-organ transplants) -Exclude pregnant patients -Exclude patients with known malignancy -Dd-cfDNA testing should not be performed within 24 h of EMB -Most cohort studies included subjects at low rejection risk (only a single center substudy of D-OAR included patients at elevated AMR risk)	-Different test thresholds can be chosen to maximize either sensitivity or specificity per the clinicians’ needs -Dd-cfDNA elevation is not specific to rejection and the assays cannot discriminate AMR from ACR, hence EMB is needed for diagnosis and to guide therapy -Assays have not been validated in European cohorts of HT recipients -Adoption of this assay in Europe faces many challenges, including cost, creation of local laboratory infrastructure with good inter-laboratory reproducibility, and obtaining approval by local regulatory agencies

The hallmark of allograft rejection is immune-mediated cell necrosis. Transplantation introduces genomic admixture with donor and recipient genomes. During allograft rejection, cell-free DNA fragments are released into the recipient’s bloodstream from the donor allograft. Leveraging transplant genomic admixture, the dd-cfDNA fraction can be identified with modern genomic techniques, which may serve as a biomarker of allograft injury and rejection. Similarly, in heart transplantation, troponin as marker of injury, and BNP as marker of graft dysfunction, may be detected in the recipient’s circulation.

On the other hand, the GEP of circulating peripheral blood mononuclear cells (PBMCs) is thought to reflect host immune responses towards the allograft, which could thus also serve as a biomarker for rejection surveillance.

In order to be useful for accurate rejection surveillance in clinical practice, a biomarker should ideally have the following characteristics: minimally invasive (blood based), quick turn-around time, good inter-sample and inter-laboratory reproducibility, affordable, accessible (in terms of technology and staff requirements), high negative predictive value (NPV) for rejection monitoring, able to categorize common transplant complications, such as acute cellular rejection (ACR), antibody-mediated rejection (AMR) and infection, and not influenced by patient or treatment factors.

In the consensus statements, available evidence on these potential biomarkers is summarized and recommendations are made on the use of non-invasive biomarkers for cardiothoracic transplant rejection surveillance.

## Methods

This consensus document follows a process that has been organized and supervised by a dedicated ESOT guidelines taskforce as outlined in a dedicated guideline [4].

Using the PICO (Population, Intervention, Comparison, Outcome) model, clinical questions were formulated, around which the expert panel’s recommendations are focused (Table 2). The rationale for the PICO questions is based on the need to provide guidance on three general domains: 1. Diagnosis/surveillance of acute rejection 2. Diagnosis/surveillance of chronic rejection 3. Prognostic stratification.

**TABLE 2:** Summary of the PICO questions and recommendations.

	PICO question		Recommendation	Level of evidence
1	*In heart transplant patients with stable graft function, are GEP and dd-cfDNA reliable surveillance tools for subclinical acute rejection monitoring, compared to endomyocardial biopsy?*	GEP	Strong for	Moderate
		Cf-ddDNA	Weak for	Low
2	*In heart transplant patients, are dd-cfDNA and GEP reliable methods to monitor for cardiac allograft vasculopathy as compared with standard diagnostic methods?*		Weak against	Very Low
3	*In heart transplant patients with stable graft function, is dd-cfDNA or GEP a reliable marker to stratify prognosis as compared to standard clinical classifiers?*		Weak against	Very Low
4	*In heart transplant patients with stable graft function, are cardiac biomarkers (NT-pro BNP, BNP, troponin) reliable surveillance tools for subclinical acute rejection monitoring, compared to EMB?*	Troponin	Weak neutral	Very Low
		BNP	Weak against	Very Low
5	*Is dd-cfDNA a reliable marker to diagnose/monitor a) clinical and subclinical acute rejection or b) infection of the graft in lung transplant patients, compared with standard diagnostic methods (surveillance bronchoscopy with TBB for histopathology and bronchoalveolar lavage for microbiology testing)?*	5A	Weak for	Low
		5B	Weak for	Very Low
6	*Is dd-cfDNA a reliable therapeutic marker to monitor treatment response for acute rejection or infection of the graft in lung transplant patients, compared with standard diagnostic methods (i.e., follow-up surveillance TBBx)?*		Weak against	Very Low
7	*Is dd-cfDNA a reliable marker to stratify prognosis of lung transplant recipients for chronic lung allograft dysfunction (CLAD), as compared to standard clinical classifiers?*		Weak for	Very Low

For each question, bibliographic searches were developed by experienced staff from the working group. Different members of the group drafted each chapter, which was then reviewed by the whole working group. The panel convened on 13–15th November 2022 (in conjunction with the ESOT TLJ 3.0 meeting in Prague and virtually), when a draft of the final recommendations with supporting evidence was presented and discussed, with further subsequent refinements.

Recommendations were graded according to the strength of the recommendation [strong (1) or weak (2)] and the quality of the evidence [high (A), moderate (B), low (C) or very low (D) (2)].

Other emerging biomarkers are briefly described below as an overview of the scientific landscape and the pipeline of discovery.

## PICO Questions and Recommendations

### Heart Transplantation

**Question 1A. In heart transplant patients with stable graft function, is GEP a reliable surveillance tool for subclinical acute rejection monitoring, compared to endomyocardial biopsy?**

**Recommendation:** Peripheral blood GEP assay (marketed in United States as Allomap®) is a reliable non-invasive diagnostic tool to rule out acute cellular rejection in stable, low-risk heart transplant recipients >15 years of age who are >55 days post HT.

• Level of evidence—moderate
• Strength of recommendation—Strong for

**Warnings:** This test is currently unavailable for clinical use in Europe.

### Supporting Evidence

The Allomap® test by CareDx Inc., United States, utilizes GEP of PBMCs to reflect host responses towards the target organ (Table 1).

Randomized studies have shown non-inferiority of Allomap-based surveillance compared to traditional biopsy-based approaches. The IMAGE study defined an abnormal score as ≥34 for adult heart transplant recipients 6 months to 5 years post-transplant, allowing to substantially reduce the number of surveillance EMBs performed, and the eIMAGE study confirmed Allomap non-inferiority in the earlier post-transplant period (55–185 days) [5, 6]. The main limitation of these studies, however, was the very low number of biopsy-proven AR.

Previously, Allomap had been rigorously validated in large observational studies, including Cardiac Allograft Rejection Gene Expression Observational (CARGO II), which included 499 heart transplant recipients from 17 predominantly European centers, and the Outcomes AlloMap Registry (OAR), which included 1,504 subjects from 35 US centers [7, 8]. Both studies showed non-inferiority of Allomap as compared to EMBs for ACR monitoring up to 5 years post-transplant, with respect to the composite outcome of rejection, graft dysfunction, death or re-transplantation, with robust NPV (>98%) and modest PPV (4%–7% among studies) [7, 8]. The OAR study additionally showed no association between higher Allomap scores and CAV, cancer or non-cytomegalovirus infection [8]. Furthermore, GEP scores did not differ between dual organ and heart alone recipients, but there are no randomized trials testing Allomap’s performance in the setting of multi-organ transplantation [8].

Allomap has received endorsement in the 2023 International Society of Heart and Lung Transplantation (ISHLT) guidelines for the care of heart transplant recipients, where it was given a class IIa, level B recommendation for ACR surveillance [9].

### Caveats

The Allomap algorithm was developed about 20 years ago, when the diagnosis and surveillance of AMR was not standard clinical practice. Implementation in Europe has been limited by cost considerations and the need to establish laboratory infrastructure for testing; as such, this test is currently unavailable for clinical use. The strength of recommendation is based on the robustness of evidence of consistent high NPV in two randomized clinical trials, in which, however, ACR was detected in less than 4% of all EMBs.

**Question 1B. In heart transplant patients with stable graft function, is dd-cfDNA a reliable surveillance tool for subclinical acute rejection monitoring, compared to endomyocardial biopsy in stable recipients?**

**Recommendation:** Beyond 4 weeks after transplantation, in addition to routine clinical care, dd-cfDNA measurements could be used to rule out clinical and subclinical rejection*,* given its high NPV.

• Level of evidence: low
• Strength of recommendation: weak for

**Warning:** Current data are based on centralized laboratory analyses; therefore, caution should be used when using assays performed in local laboratories.

### Supporting Evidence

DD-cfDNA assays for rejection surveillance have never been compared head-to-head with EMBs in a randomized clinical trial. The current evidence for their utility in AR monitoring comes primarily from three large cohort studies that were conducted mainly in the United States—the GRAfT study used a research-grade assay, D-OAR used Allosure (CareDx) and the DEDUCE study employed Prospera Heart (Natera). Several dd-cfDNA assays by different vendors have been developed (Table 1).

The GRAfT study used a threshold of ≥0.25% and showed that the test had sensitivity 81%, specificity 85%, PPV 19.6%, NPV 99.2% for rejection detection, defined as ACR≥2R and pAMR ≥1 [3]. The study demonstrated that using a dd-cfDNA-based monitoring strategy could have safely avoided 81% of all routine EMBs [3]. Allosure has been validated in a US-based prospective observational cohort study (D-OAR) of 740 heart transplant recipients in the first 2 years post HT(10). At a 0.2% threshold, the test had sensitivity 44%, specificity 80%, PPV 8.9% and NPV 97.1% to differentiate AR from no rejection [10]. The D-OAR study also included a parallel arm single-center cohort of 33 heart transplant recipients at high risk for AMR and showed that the test had similar performance in this group [10].

The DEDUCE study was an observational 2-center study with retrospective and prospective components testing the performance of the Prospera dd-cfDNA assay for AR surveillance [11]. It included 811 samples from 223 heart transplant recipients [11]. Using a proposed threshold of ≥0.15% for the assay, it had sensitivity 79%, specificity 77%, PPV 25% and NPV 97% for AR. In biopsy-matched non-rejection samples the dd-cfDNA fraction was stable up to 24 months post-transplant, and increased after 24 months [11].

The FreeDNA-CAR was an observational study including 206 patients from 12 Spanish transplant centers. By using the Allonext^®^ assay (Eurofins Genome) a threshold of ≥0.15% had a 97% NPV for AR. This study was presented at the ESOT 2023 congress and is not yet available as peer reviewed publication.

### Caveats and Unmet Needs

Different dd-cfDNA thresholds have been tested in available studies, ranging from 0.15% to 0.25%. Assay variability, limit of detection, and other characteristics vary between commercially available tests, and the rate of rejection in the study populations may affect the resulting test performance. These test characteristics, in addition, have been determined in studies with centralized laboratory measurements. It is unknown how they might be applicable in clinical practice when the assay is performed in local laboratories.

It must be noted that, as compared to kidney and lung transplantation studies, the dd-cfDNA threshold value is much lower in heart transplantation. An important question which remains unaddressed is whether the accuracy of the dd-cfDNA test, often reported as coefficient of variability, is applicable at different dd-cfDNA thresholds. However, even with constant standard variation, the coefficient of variability of an assay is inversely related to the mean value at different ranges of the data. In addition, variability of assay measures generally increases with lower concentration of the analyte. Thus, a coefficient of variability measured at high dd-cfDNA thresholds used for lung and kidney transplantation may not be applicable in heart transplantation with lower dd-cfDNA thresholds. The coefficient of variability should be computed around all desired thresholds.

Some data suggest that absolute dd-cfDNA quantity may be a better marker than dd-cfDNA fraction, as it is independent of changes in background (recipient) cfDNA levels. In the DEDUCE study, a *post-hoc* analysis using dd-cfDNA quantity indicated that incorporation of this measure could increase the sensitivity of the assay [11].

There is a paucity of data on whether dd-cfDNA assays can be employed for monitoring treatment response during and after AR. Small studies have shown a reduction in cfDNA levels after rejection treatment; however, the assays have not been validated for therapeutic guidance. Additionally, dd-cfDNA levels have been shown to be elevated in patients with *de novo* donor-specific antibodies (DSAs), raising the possibility of identifying pathological DSAs using these assays. However, these preliminary findings are hypothesis-generating and must be verified in large studies.

**Question 2. In heart transplant patients, are dd-cfDNA and GEP reliable methods to monitor for cardiac allograft vasculopathy as compared with standard diagnostic methods?**

**Recommendation:** We do not recommend the use of either dd-cfDNA or GEP (Allomap) as surveillance strategies for cardiac allograft vasculopathy post-heart transplantation.

• Level of evidence: very low
• Strength of recommendation: weak against

### Supporting Evidence

A small single-center pilot study performed in the US showed that dd-cfDNA is elevated in cardiac allograft vasculopathy (CAV) and suggested endothelial injury and ischemia as possible mechanisms [12]. However, another study from Spain using different dd-cfDNA detection methods and thresholds did not confirm the association between dd-cfDNA and CAV [13]. These divergent findings underscore the need for larger prospective studies to define the role of dd-cfDNA in screening for CAV (the SHORE registry is exploring this question). Similarly, a retrospective study did not support the use of GEP for CAV surveillance [8].

**Question 3. In heart transplant patients with stable graft function, is dd-cfDNA or GEP a reliable marker to stratify prognosis as compared to standard clinical classifiers?**

**Recommendation:** We do not suggest the use of dd-cfDNA or GEP to stratify prognosis after heart transplantation, despite several studies showing associations of these biomarkers with long-term clinical events

• Level of evidence: Very low
• Strength of recommendation: Weak against

### Supporting Evidence

There are no studies specifically powered and designed for exploring the prognostic role of either GEP or dd-cfDNA in heart transplantation. Major studies on these biomarkers were performed in stable low-risk patients, with very low mortality rates during their limited follow-up (up to 3-year) [7, 14]. Moreover, the few available post-hoc analyses reporting combined clinical outcomes provide contradictory results.

No association has been found between GEP scores and mortality during follow-up in different studies. Two sub-studies of major trials (IMAGE [15] and CARGO II [7]) published by Deng et al in 2014 [16] and Crespo-Leiro et al in 2015 [17], tested the performance of AlloMap™ as a predictor of major adverse cardiac transplant events (MACTE, a composite of acute rejection with hemodynamic compromise, graft dysfunction, death or retransplantation). In both cases, intraindividual variability (standard deviation of ≥4 GEP scores) predicted a higher incidence of MACTE in the next 2-3 years, with a hazard ratio of 1.76 per unit increase in variability in one of the studies [16]. Other ways of measuring repeated individual GEP scores (ordinal score, scores above a given threshold) did not show a similar predictive ability. Moreover, in the OAR study (Moayedi 2019) [8], no meaningful changes in GEP were seen in relation to specific heart transplant complications such as CAV, cancer or non-cytomegalovirus infections.

However, the existence of 2 sub-studies [16, 17] of major GEP trials with reasonably sized populations (369 and 91 patients, respectively) and differing characteristics (one USA-based, the second mainly European) with coincidental findings should not be dismissed to potentially identify GEP score change as a predictor of adverse outcomes. The main limitation of these studies is the need for ≥4 consecutive GEP scores to evaluate variability (standard deviation of all scores).

As for dd-cfDNA, a preliminary study (Zangwill, 2020) [18] focused on the first 10 days after heart transplantation in a small pediatric population showed that a blunted decline of initially elevated dd-cfDNA may be associated with early death. Two other studies found that total cfDNA levels greater than 50 ng/mL were associated with increased risk of major events (composite outcome of cardiac arrest, mechanical circulatory support, death) (Zangwill 2022 [19]), death (Scott, Zangwill 2022 [19, 20]) and treatment for infection [19, 20].

Only one exploratory abstract (Crespo-Leiro, 2017) [21] has been directed to evaluate the prognostic value of dd-cfDNA in stable HT recipients. It included 48 patients and 166 samples from the CARGO-II trial, and showed an association between the median of several individual dd-cfDNA values and subsequent incidence of MACTE (as defined above), *p* = 0.02, AUCOR = 0.77. Other dd-cfDNA measures, such as maximum value, individual measures, or variability of intraindividual measures did not predict MACTE.

Of note, several groups have found clear relationships between “total or nuclear cfDNA” (derived both from recipient and donor tissues) and several near-term events, such as death, cardiac arrest, and need for mechanical circulatory support [19]. Total cfDNA seems to be a marker of more extensive tissue damage, and has demonstrated prognostic value in different ICU patient populations. Total cfDNA elevations have also been seen in patients with infections after heart transplantation [20]. The same is true for sepsis, inflammatory diseases and cancer in non-transplant populations.

### Caveats and Unmet Needs

Despite current available data do not support the use of dd-cfDNA as a biomarker predictive for subsequent clinical events, in the GRAfT study [3] dd-cfDNA elevations associated with negative EMB were predictive of subsequent biopsy-proven AR or allograft dysfunction. These findings suggest that asymptomatic dd-cfDNA elevation represents an opportunity for additional testing (e.g., donor-specific antibodies) and early intervention prior to detection of histopathological rejection. Current data do not support use of dd-cfDNA to titrate immunosuppressive medications, but the above preliminary findings suggest that patients with elevated dd-cfDNA in the absence of biopsy-proven rejection may benefit from closer monitoring. It remains to be investigated if intensification of immunosuppression in the setting of elevated dd-cfDNA and absence of histologic rejection could mitigate future episodes of biopsy-proven rejection, graft injury and/or graft dysfunction. On the other hand, we may hypothesize that low dd-cfDNA levels can be used to guide safe weaning of immunosuppression, thus decreasing lifelong risks of infections, malignancies and renal dysfunction, among complications. HeartCare Immuno-optimization in Cardiac Allografts (MOSAIC) (NCT05459181) is one such study aimed to determine whether patients at low risk of acute rejection can safely wean their post-transplant immunosuppressive medications using a combination of tests that include DSA, histology, donor-derived cell-free DNA (AlloSure), and gene expression profiling (AlloMap). The study is in the planning stages and is designed as an unblinded randomized controlled study of 930 HT recipients enrolled within 2 weeks of HT.

**Question 4. In heart transplant patients with stable graft function, are cardiac biomarkers (NT-pro BNP, BNP, troponin) reliable surveillance tools for subclinical acute rejection monitoring, compared to EMB?**

**Recommendation 4.A:** There is inadequate evidence to support the routine use of cardiac troponin (or high-sensitivity troponin) for the diagnosis of AR after heart transplantation, due to conflicting data.

• Level of evidence: very low
• Strength of recommendation: weak neutral

### Supporting Evidence

Cardiac troponin (cTn) is the hallmark biomerker of cardiac damage and bears a central role in general cardiology for the diagnosis of acute coronary syndromes and to stratify cardiovascular prognosis. However its role in the setting of heart transplantation is controversial. Myocyte damage is the pathologic hallmark of moderate to severe ACR, so an elevated cTn level would be expected during an episode of ACR, in particular for high-sensitivity assays (hs-cTn) [22–24]. However, the results of different studies are conflicting, with some reporting no association between cTn and EMB-proven ACR [24–26] and others finding that cTn levels [27] were significantly higher in patients with ACR [28–32].

A systematic review with meta-analysis of 27 studies with 1,684 patients confirmed a poor diagnostic accuracy [33].

*A systematic review by Fitzsimons et al* [34] *showed that cTn assays did not have sufficient specificity to diagnose ACR in place of EMB, but hs-cTn assays may have sufficient sensitivity and negative predictive value to exclude ACR and limit the need for surveillance EMB.*

### Caveats and Unmet Needs

Studies about cTn in the diagnosis or surveillance of rejection are mostly small-sized, retrospective and single center, leading to conflicting results. No randomized or prospective observational multicenter studies are available. Nevertheless, given the universal availability and low cost of the assay, and the proven reliability of this biomarker for cardiac injury, it may provide support to the complete clinical evaluation in ruling out acute cardiac injury in stable patients.

**Recommendation 4.B:** We do not suggest the routine use of natriuretic peptides (BNP, NT-pro BNP) to monitor for subclinical AR in stable heart transplant patients, due to the many clinical factors that can affect BNPs levels.

• Level of evidence: very low
• Strength of recommendation: weak against

### Supporting Evidence

Natriuretic peptides (NPs) are hormones produced by the myocardium in response to atrial and ventricular wall stress. BNP and its pro-hormone NT-proBNP are widely used in the diagnosis and prognostic stratification of heart failure patients. These biomarkers are sensitive to treatment and have also been used as surrogate endpoints for drug efficacy. Despite the fact that they are widely studied in the context of heart failure, evidence in the setting of heart transplantation is sparse and of poor quality.

Most observational studies showed that BNP/NT-proBNP levels were significantly higher in patients with graft rejection [32, 35–39]; however, they had low discriminating power to detect clinically significant episodes of rejection. There was a considerable overlap in BNP/NT-proBNP levels in patients with and without significant ACR.

BNP levels are reported to be higher in heart transplant recipients than in the general population, and are sensitive to higher grades of rejection and left ventricular dysfunction [35]. Klingenberg et al observed that changes in BNP levels compared to baseline were more useful, as BNP values could be influenced by patient variables such as sex or renal function, or transplant variables such as post-transplant time [40]. The association of BNP with AR and the usefulness of serial measurements were corroborated by other studies [41–43]. Prior studies have also demonstrated a decrease in NP levels in the first 6 months after transplant, which then reach a plateau [44, 45]. NP levels have further been shown to correlate with allograft dysfunction, cardiac allograft vasculopathy and cardiovascular death [46, 47].

However, other studies have found that BNP levels lack sufficient discriminatory ability to guide the performance of EMBs [48, 49]. In summary, despite initial promising studies, later studies did not find any association between AR episodes and BNP [50] or NT-proBNP [25, 51].

### Caveats and Unmet Needs

The low quality of available evidence, the heterogeneity of factors affecting NP levels, and the conflicting results of published studies do not support the use of NPs for non-invasive surveillance of acute rejection. However, high levels of NPs are associated with poor long-term post-transplant prognosis, and in the context of multiparametric clinical evaluation, NP levels may help guide the assessment of graft function in asymptomatic patients.

### Lung Transplantation

**Question 5. Is dd-cfDNA a reliable marker to diagnose/monitor a) clinical and subclinical acute rejection or b) infection of the graft in lung transplant patients, compared with standard diagnostic methods (surveillance bronchoscopy with TBB for histopathology and bronchoalveolar lavage for microbiology testing)?**

**Recommendations:**

A) Beyond 6 weeks of transplantation, in addition to routine clinical care, dd-cfDNA measurements could be used to rule out clinical and subclinical rejection, given its high NPV for rejection diagnosis.

• Level of Evidence = low
• Strength of recommendation = weak for

B) Beyond 6 weeks of transplantation, in addition to routine clinical care, dd-cfDNA measurements could be used to rule out infection.

• Level of Evidence = very low
• Strength of recommendation = weak for

### Supporting Evidence

In cohort studies, dd-cfDNA increased with histologically documented ACR and clinical AMR [14, 52–59] The cohort studies reported good test performance of dd-cfDNA with a high NPV to detect rejection. Indeed, levels of dd-cfDNA increased up to 2–4 months prior to the diagnosis of AMR [52, 57].

Some studies reported [14, 54] that dd-cfDNA also increased in patients with infections, while other studies found no correlation [55, 58, 59]. While dd-cfDNA levels were often similar between pathogen positive and pathogen negative timepoints across lung transplant studies, two studies examined the association between the presence of pathogens with or without concomitant infectious symptoms and dd-cfDNA levels (infection was defined as detection of pathogens plus a reduction in pulmonary function test or presence of pulmonary symptoms. The studies showed higher dd-cfDNA levels at infection compared to stable controls or pathogens without signs or symptoms of infection; levels were similar for infection and acute rejection [52, 58].

For patients with serial dd-cfDNA levels and with dd-cfDNA levels <1%, fluctuations, increases in dd-cfDNA from baseline or less are normal [60]. From one multicenter study, monthly dd-cfDNA was used in routine care for surveillance for acute lung allograft dysfunction (ALAD). In total 175 patients were enrolled and followed over 6 months. A 1% dd-cfDNA level was used as a rule out threshold with a sensitivity of 74%, specificity of 88%, a PPV of 43% and NPV of 97% to detect ALAD, a composite endpoint of infection and acute rejection [58].

### Optimal dd-cfDNA Thresholds and Relevance

Considerations in selecting a dd-cfDNA threshold as a rule out test include the test characteristic being prioritized (sensitivity, specificity, and PPV and NPV) or whether the patient has a single versus double lung transplant.

In the GRAfT and ALARM Studies, defining acute rejection as histopathology ACR grade ≥2 or histopathology grade 1 plus a reduction in forced expiratory volume in one second (FEV1) by at least 10% or presence of pulmonary symptoms and/or clinical AMR defined by the 2016 International Society for Heart and Lung Transplantation Consensus criteria, a 1% dd-cfDNA threshold showed sensitivity of 74%–77%, specificity of 84%–88%, NPV of 90%–97% and PPV of 43%–64% [52, 58]. In one study, defining acute rejection as only grade 3 and 4 ACR, a 1% dd-cfDNA showed a sensitivity of 100% [53]. These studies did not differentiate clinical from subclinical acute rejection.

In the GRAfT Study, a lower threshold of dd-cfDNA, 0.5%, showed sensitivity of 95%, specificity of 65% and NPV of 96% and a PPV of 64% [3]. In two other studies, a 0.85% or 0.87% threshold showed sensitivity of 76% and 73%, specificity of 53% and 56%, PPV of 34% and 43% and NPV of 84% and 86%, respectively [55, 59].

The optimal dd-cfDNA threshold for detection of acute rejection is lower in single lung (0.54%) vs. double lung transplant (1.1%): differences in dd-cfDNA in single versus double lung transplant is key for the interpretation of dd-cfDNA testing in research and clinical settings [56].

### Timing of Initiation of Surveillance With dd-cfDNA Monitoring

We suggest use of dd-cfDNA starting from week 6 of transplantation and until 18–24 months after transplant. In the GRAfT Study, dd-cfDNA levels are high after transplant surgery, followed by a decay to reach low stable levels by week 6. Levels remain stable thereafter and increased beyond 2 years of transplant [52]. Stable, asymptomatic patients, independent of the risk of rejection or infection may be considered for dd-cfDNA assay. Studies thus far include adult transplant patients only.

### Caveats and Knowledge Gaps

Several caveats and knowledge gaps still exist in the validation process for clinical use of dd-cfDNA in lung transplantation. The major areas of uncertainty are summarized in Table 3.

**TABLE 3:** Caveats regarding interpretation of dd-cfDNA in lung transplant recipients.

• Donor fraction vs. absolute dd-cfDNA levels: no available data in lung transplantation. There is a need for studies to elucidate this point
• Prognostic role of asymptomatic dd-cfDNA elevation: in the GTD, ALARM and GRAfT Studies [11, 15, 16], high dd-cfDNA levels were observed up to 6 months prior to clinically significant events (graft dysfunction, pathological rejection diagnosis, etc.). This represents an opportunity for early diagnosis and treatment. However, no studies have been performed to date to determine the prognostic value of asymptomatic dd-cfDNA elevations
• dd-cfDNA for surveillance of acute rejection treatment response: studies have shown reduction in cfDNA levels with initiation of acute rejection treatment [11, 12, 15, 16]; however, the correlation of the dd-cfDNA trends and treatment response remain undefined
• dd-cfDNA assays are unable to differentiate AMR from ACR and hence, the need for TBBx (+/− more advanced gene expression testing) to determine rejection type, as this guides treatment approach. Fortunately, novel cfDNA approaches show promise, being able to target tissue-specific cfDNA to identify the cells and tissue involvement and/or identify disease molecular pathways. Perhaps these novel cfDNA approaches show improved specificity to differentiate AMR from ACR.
• dd-cfDNA assays are currently processed in central laboratories in the USA with relatively slow turn-around time of up to 72 h (Allosure and Prospera); the adoption of this technology in Europe is limited by cost considerations, regulatory approval by local agencies and the availability of the appropriate equipment and technology at local centers
• dd-cfDNA levels are affected by multi-organ transplants, active malignancy, prior bone marrow transplant, pregnancy, <24 h following an TBBx, and sepsis, for example,

**Question 6. Is dd-cfDNA a reliable therapeutic marker to monitor treatment response for acute rejection or infection of the graft in lung transplant patients, compared with standard diagnostic methods (i.e., follow-up surveillance TBBx)?**

**Recommendation:** While dd-cfDNA levels generally decline after treatment for acute rejection or infection is initiated, we currently do not suggest using dd-cfDNA as an indicator of treatment response.

• Level of evidence: very low
• Strength of recommendation: weak against

### Supporting Evidence

In observational cohort studies, dd-cfDNA levels increased with detection of acute rejection or infection. The dd-cfDNA levels generally reduced with initiation of treatment. However, the relationship of the post-treatment dd-cfDNA kinetics and treatment response has not been addressed [52, 53, 57, 58].

### Caveats and Knowledge Gaps

Carefully designed studies are needed to test if the dynamics of dd-cfDNA trends reflect response to treatment.

**Question 3. Is dd-cfDNA a reliable marker to stratify prognosis of lung transplant recipients for chronic lung allograft dysfunction (CLAD), as compared to standard clinical classifiers?**

**Recommendations:**

1. Dd-cfDNA levels and trends in the **early post-transplant period** could be used as a predictive marker for early death and/or CLAD in lung transplant patients.
  • Level of Evidence = very low
  • Level of recommendation = weak for
2. In patients with **primary graft dysfunction (PGD)**, dd-cfDNA levels could be used to predict subsequent risk of CLAD.
  • Level of Evidence = very low
  • Level of recommendation = weak for
3. For patients with **respiratory viral infections**, dd-cfDNA levels at time of infection might be used to predict subsequent risk of CLAD and/or CLAD progression.
  • Level of Evidence = very low
  • Level of recommendation = neutral

### Supporting Evidence

In a study combining the GRAfT and GTD cohorts, early post-transplant average dd-cfDNA levels, computed as the mean of at least three dd-cfDNA measurements between day 14 and 90 post-transplant could predict subsequent CLAD. Patients with average dd-cfDNA in the upper tertile showed a 6.6-fold higher risk of early death and/or CLAD and 4 times higher risk of developing AMR as compared to those in the lower tertile. A 1% increase in average dd-cfDNA increased the risk of early death/CLAD by ∼40% (HR 95% CI 1·1–1·5, *p* = 0·015) [61]. In a small pilot study, average dd-cfDNA levels were higher for patients who developed CLAD than for patients who did not develop CLAD [61].

From the same two cohorts, dd-cfDNA stratified PGD patients for subsequent risk of CLAD. Patients with PGD and high dd-cfDNA on day 3 of transplant showed increased odds of CLAD compared to patients with PGD and low dd-cfDNA levels [62].

The GRAfT study categorized pathogens based on their known risk of CLAD and showed that high-risk pathogens had higher dd-cfDNA levels at detection compared to low-risk pathogens. In patients with respiratory viral pathogens, dd-cfDNA ≥1% showed 2 times greater rates development of CLAD, CLAD stage progression and/or death, within 1 year of detection of viral pathogen [63].

### Caveats and Knowledge Gaps

All the evidence supporting these recommendations are derived from studies performed by the same research group on two cohort of patients. There is a need for well-designed studies to test the prognostic utility of dd-cfDNA levels and trends in lung transplantation with respect to risk stratification.

## Conclusion

This document provides current evidence on four known and upcoming biomarker assays and their use in rejection surveillance of cardiothoracic transplant recipients. The recommendations are aiming at optimizing clinical practice, patient health and post-transplant clinical outcome as well as identifying priorities for future research. Dd-cfDNA is the biomarker closest to the clinical applicability *in lieu* of the several observational studies showing a good negative predictive power to rule out rejection. However, the recommendation in favor of its use it is still supported by weak evidence because a prospective randomized study proving the benefit of this biomarker over the standard surveillance approaches is still lacking. An important limitation of dd-cfDNA is its low specificity. However, with this limitation and its high sensitivity, dd-cfDNA can be an ideal biomarker to monitor cardiothoracic transplant patients to rule out acute graft injury. Standard cardiac biomarkers such as troponin and natriuretic peptides cannot be recommended in standard clinical practice for rejection surveillance because of scattered and contradictory data. However, both troponin and natriuretic peptides may have a role in stratifying the prognosis and in identifying patients with subclinical graft dysfunction or injury. Additional biomarkers (Table 4) with a potential of being useful in cardiothoracic transplantation, like cfDNA epigenetic analysis and fragmentomics, exosomes, microRNA or multimodal approaches are in the pipeline but will need additional examination before implementation in clinical practice. These upcoming approaches may improve on the low specificity of dd-cfDNA to identify acute rejection phenotypes or other transplant complications.

**TABLE 4:** Emerging biomarkers in the heart transplant field.

	Description	The state of the field	Notable studies
MicroRNAs	• small non-coding RNAs • regulate mRNA translation within pathways involved in innate and adaptive immune responses [64] • very stable in the circulation • transported within exosomes, microvesicles, and apoptotic bodies	• various miRNA panels proposed in single center studies—heterogeneity likely reflects different methodologies, patient populations studied [65–67] • not validated in large prospective studies • not available for clinical use • potential for non-invasive discrimination between AMR/ACR and potential target for therapeutic interventions	• Shah et al [68]—largest study to-date with validation in an external set of samples • proposed 12 miRNAs for ACR and 17 miRNAs for AMR monitoring with converted score 0–100 • at a threshold of ≥65, the assay has 86% sensitivity, 76% specificity, and 98% NPV for ACR, and 82% sensitivity, 84% specificity and 97% NPV for AMR
Exosomes	• small extracellular vesicles (EV) released by cells into body fluids (serum, urine, etc.) [67] • modulate immune responses through communication with surface receptors on leukocytes or intracellular delivery of immune mediators	• various exosome panels have been proposed in small and primarily single center studies [67, 69–71] • need for streamlining of the process of exosome isolation and refinement of the surface marker panels • pending validation in large prospective studies • not available for clinical use	• Castellani et al [69] -largest study to date with a training and a validation cohort • used surface marker analysis by multiplex flow cytometry • according to differential EV-marker expression, a diagnostic model was built and validated in an external cohort of patients with accuracy of the model reaching 86.5% [69]
Digital Pathology	• employs computational image analysis using machine learning methodologies [72] • aims to improve EMB grading consistency and sensitivity	• in a multi-center study, computational histological analysis of digitalized EMBs had similar diagnostic concordance as expert pathologists [72]	• Peyster et al [72]—showed that adding deeper phenotyping of biopsy tissue using quantitative multiplexed immunofluorescence techniques improves the diagnostic and prognostic performance of histologic analysis for AR. • Peyster et al [73]- a CAV prediction model was built combining clinical variables with morphological features from digitized EMB samples, and the model accurately identified patients at risk for CAV development years prior to the disease onset.
Intragraft gene expression profiling	2 types of assays • genome-wide microarray analysis of mRNA transcripts (MMDx^®^ Heart) [74]—uses machine learning algorithms to assign samples to 4 archetypes (normal, Amr, ACR, injury); each new sample enriches the reference set, thus propagating the learning and development loop of this technique. Requires fresh EMB sample • restricted gene expression signatures (nCounter^®^) [75]—allow the exploration of a limited number of transcripts from formalin-fixed paraffin-embedded tissue, thus conducive to longitudinal studies	• development of MMDx®-Heart was initially based on 331 EMB samples and identified 3 archetypes: ACR, AMR and no rejection. The study reported AUCs of 0.78 (no rejection), 0.65 (ACR), and 0.81 (AMR) [76] • the ongoing 13-center INTERHEART study (NCT02670408) continues to validate and refine this system. Proposes that injury is more important prognostically than AR histological grade for the outcomes of long-term graft survival [75] • single center analysis reports 61% concordance among MMDx^®^, dd-cfDNA and EMB histopathology for AR; 84% agreement reported between EMB and MMDx^®^ [77] • The MMDx^®^ Heart is now commercially available and can be clinically applied • nCounter^®^ is in early stages of development and validation	• the Trifecta-Heart cfDNA-MMDx study (NCT04707872) proposes to calibrate dd-cfDNA levels (using Natera’s Prospera^®^ assay) obtained at the time of a for-cause or protocol biopsy against the MMDx measurements as a new proposed gold standard
Cardiac magnetic resonance imaging	• multiparametric tissue and functional characterization -T2 mapping for myocardial edema, pre- and post-gadolinium contrast T1 mapping to quantify extracellular volume fraction [78] • assesses global cardiac structure and function and regional tissue characteristics that can capture patchy areas of AR not detected on EMB [78]	• available for clinical use • earlier studies used older spin echo sequences and showed inconsistent results in the detection of AR; with multi-parametric imaging, CMR has been shown to have good diagnostic performance in small studies and 1 single-center randomized trial [79, 80] • CMR requires high level of technical expertise and T1, T2 values can vary widely with magnet field strength, sequencing protocol used or machine specifications • lack of long-term outcomes data for a CMR-based surveillance protocol • ISHLT 2022 guidelines assign Class IIb, Level of Evidence C recommendation for its use as an adjunct modality in patients with unexplained graft dysfunction and low-grade or absent histologic evidence of rejection on EMB	• Anthony et al [81]—cross-sectional observational study showed CMR had sensitivity 93%, specificity 92%, NPV 99% and PPV of 62% for AR • Anthony et al [81] – single center trial that randomized 40 HT recipients at 4 weeks post-transplant to either conventional EMB-based or CMR-based surveillance. The 2 groups had similar rates of ≥2R rejection and mortality at 1yr. CMR-based surveillance led to a substantial reduction in the number of EMBs (by 94%) as well as unplanned hospitalizations

ACR, acute cellular rejection; AMR, acute antibody mediated rejection; AR, acute rejection, Cav, cardiac allograft vasculopathy; CMR, cardiac magnetic resonance; EMB, endomyocardial biopsy; HT, heart transplant; ISHLT, the International Society of Heart and Lung Transplantation; MMDx, Molecular Microscope; NPV, negative predictive value; PPV, positive predictive value.

## References

[1] Crespo-Leiro MG Zuckermann A Bara C Mohacsi P Schulz U Boyle A Concordance Among Pathologists in the Second Cardiac Allograft Rejection Gene Expression Observational Study (CARGO II). Transplantation (2012) 94(11):1172–7. 10.1097/TP.0b013e31826e19e2 23222738

[2] Lund LH Khush KK Cherikh WS Goldfarb S Kucheryavaya AY Levvey BJ The Registry of the International Society for Heart and Lung Transplantation: Thirty-Fourth Adult Heart Transplantation Report—2017; Focus Theme: Allograft Ischemic Time. J Heart Lung Transpl (2017) 36(10):1037–46. 10.1016/j.healun.2017.07.019 28779893

[3] Agbor-Enoh S Shah P Tunc I Hsu S Russell S Feller E Cell-Free DNA to Detect Heart Allograft Acute Rejection. Circulation (2021) 143(12):1184–97. 10.1161/CIRCULATIONAHA.120.049098 33435695 PMC8221834

[4] Cillo U Weissenbacher A Pengel L Jochmans I Roppolo D Amarelli C ESOT Consensus Platform for Organ Transplantation: Setting the Stage for a Rigorous, Regularly Updated Development Process. Transpl Int (2022) 35:10915. 10.3389/ti.2022.10915 36406781 PMC9667481

[5] Pham MX Teuteberg JJ Kfoury AG Starling RC Deng MC Cappola TP Gene-Expression Profiling for Rejection Surveillance after Cardiac Transplantation. N Engl J Med (2010) 362(20):1890–900. 10.1056/NEJMoa0912965 20413602

[6] Kobashigawa J Patel J Azarbal B Kittleson M Chang D Czer L Randomized Pilot Trial of Gene Expression Profiling versus Heart Biopsy in the First Year after Heart Transplant: Early Invasive Monitoring Attenuation through Gene Expression Trial. Circ Heart Fail (2015) 8(3):557–64. 10.1161/CIRCHEARTFAILURE.114.001658 25697852

[7] Crespo-Leiro MG Stypmann J Schulz U Zuckermann A Mohacsi P Bara C Clinical Usefulness of Gene-Expression Profile to Rule Out Acute Rejection after Heart Transplantation: CARGO II. Eur Heart J (2016) 37(33):2591–601. 10.1093/eurheartj/ehv682 26746629 PMC5015661

[8] Moayedi Y Foroutan F Miller RJH Fan CS Posada JGD Alhussein M Risk Evaluation Using Gene Expression Screening to Monitor for Acute Cellular Rejection in Heart Transplant Recipients. J Heart Lung Transpl (2019) 38(1):51–8. 10.1016/j.healun.2018.09.004 30352779

[9] Velleca A Shullo MA Dhital K Azeka E Colvin M DePasquale E The International Society for Heart and Lung Transplantation (ISHLT) Guidelines for the Care of Heart Transplant Recipients. J Heart Lung Transpl (2023) 42(5):e1–141. 10.1016/j.healun.2022.10.015 37080658

[10] Khush KK Patel J Pinney S Kao A Alharethi R DePasquale E Noninvasive Detection of Graft Injury after Heart Transplant Using Donor-Derived Cell-Free DNA: A Prospective Multicenter Study. Am J Transpl (2019) 19(10):2889–99. 10.1111/ajt.15339 PMC6790566 30835940

[11] Kim PJ Olymbios M Siu A Wever Pinzon O Adler E Liang N A Novel Donor-Derived Cell-Free DNA Assay for the Detection of Acute Rejection in Heart Transplantation. J Heart Lung Transpl (2022) 41(7):919–27. 10.1016/j.healun.2022.04.002 PMC9670834 35577713

[12] Holzhauser L Clerkin KJ Fujino T Alenghat FJ Raikhelkar J Kim G Donor-Derived Cell-Free DNA Is Associated with Cardiac Allograft Vasculopathy. Clin Transpl (2021) 35(3):e14206. 10.1111/ctr.14206 PMC10040222 33368611

[13] Jiménez-Blanco Bravo M Pérez-Gómez L Hernández-Pérez FJ Arellano-Serrano C Torres-Sanabria M Gómez-Bueno M Lack of Usefulness of Donor-Derived Cell-Free DNA as a Biomarker for Cardiac Allograft Vasculopathy: A Prospective Study. Front Cardiovasc Med (2022) 9:856600. 10.3389/fcvm.2022.856600 35463750 PMC9019134

[14] Sorbini M Togliatto G Mioli F Simonato E Marro M Cappuccio M Validation of a Simple, Rapid, and Cost-Effective Method for Acute Rejection Monitoring in Lung Transplant Recipients. Transpl Int (2022) 35:10546. 10.3389/ti.2022.10546 35755857 PMC9221674

[15] Pham MX Deng MC Kfoury AG Teuteberg JJ Starling RC Valantine H. Molecular Testing for Long-Term Rejection Surveillance in Heart Transplant Recipients: Design of the Invasive Monitoring Attenuation through Gene Expression (IMAGE) Trial. J Heart Lung Transpl (2007) 26(8):808–14. 10.1016/j.healun.2007.05.017 17692784

[16] Deng MC Elashoff B Pham MX Teuteberg JJ Kfoury AG Starling RC Utility of Gene Expression Profiling Score Variability to Predict Clinical Events in Heart Transplant Recipients. Transplantation (2014) 97(6):708–14. 10.1097/01.TP.0000443897.29951.cf 24637869 PMC3983476

[17] Crespo-Leiro MG Stypmann J Schulz U Zuckermann A Mohacsi P Bara C Performance of Gene-Expression Profiling Test Score Variability to Predict Future Clinical Events in Heart Transplant Recipients. BMC Cardiovasc Disord (2015) 15(1):120. 10.1186/s12872-015-0106-1 26452346 PMC4600291

[18] Zangwill SD Kindel SJ Ragalie WS North PE Pollow A Hidestrand M Early Changes in Cell‐free DNA Levels in Newly Transplanted Heart Transplant Patients. Pediatr Transpl (2020) 24(1):e13622. 10.1111/petr.13622 PMC7065377 31825144

[19] Zangwill SD Deshpande SR Simpson PM Liang HL Zhang L Dasgupta M Increase in Nuclear Cell-Free DNA Is Associated with Major Adverse Events in Adult and Pediatric Heart Transplant Recipients. Clin Transpl (2022) 36(1):e14509. 10.1111/ctr.14509 34649304

[20] Scott JP Ragalie WS Stamm KD Mahnke DK Liang HL Simpson PM Total Cell-Free DNA Predicts Death and Infection Following Pediatric and Adult Heart Transplantation. Ann Thorac Surg (2021) 112(4):1282–9. 10.1016/j.athoracsur.2020.08.006 33039362

[21] Crespo-Leiro M Hiller D Woodward R Grskovic M Marchis C Song M Analysis of Donor-Derived Cell-Free DNA with 3-Year Outcomes in Heart Transplant Recipients. J Heart Lung Transpl (2017) 36(4):S69–70. 10.1016/j.healun.2017.01.172

[22] Hill DA Drazner MH De Lemos JA. Do Established Biomarkers Such as B-Type Natriuretic Peptide and Troponin Predict Rejection? Curr Opin Organ Transpl (2013) 18(5):581–8. 10.1097/MOT.0b013e328364fe23 23995368

[23] Erbel C Taskin R Doesch A Dengler TJ Wangler S Akhavanpoor M High-Sensitive Troponin T Measurements Early after Heart Transplantation Predict Short and Long-Term Survival. Transpl Int (2013) 26(3):267–72. 10.1111/tri.12024 23252662

[24] Ahn KT Choi JO Lee GY Park HD Jeon ES. Usefulness of High-Sensitivity Troponin I for the Monitoring of Subclinical Acute Cellular Rejection after Cardiac Transplantation. Transpl Proc (2015) 47(2):504–10. 10.1016/j.transproceed.2014.10.049 25769598

[25] Battes LC Caliskan K Rizopoulos D Constantinescu AA Robertus JL Akkerhuis M Repeated Measurements of NT-pro-B-type Natriuretic Peptide, Troponin T or C-Reactive Protein Do Not Predict Future Allograft Rejection in Heart Transplant Recipients. Transplantation (2015) 99(3):580–5. 10.1097/TP.0000000000000378 25136844

[26] Mullen JC Bentley MJ Scherr KD Chorney SG Burton NI Tymchak WJ Troponin T and I Are Not Reliable Markers of Cardiac Transplant Rejectionq. Thorac Surg (2002). 10.1016/s1010-7940(02)00293-2 12142191

[27] Wahlander H Kjellstrom C Holmgren D. Sustained Elevated Concentrations of Cardiac Troponin T during Acute Allograft Rejection after Heart Transplantation in Children1. Transplantation (2002) 74(8):1130–5. 10.1097/00007890-200210270-00013 12438959

[28] Gleissner CA Zehelein J Sack FU Schnabel P Haass M Dengler TJ. Extended Experience and Subgroup Analysis Using Cardiac Troponin T for Rejection Monitoring after Heart Transplantation. Transpl Proc (2002) 34(6):2178–80. 10.1016/s0041-1345(02)03194-9 12270356

[29] Balduini A Campana C Ceresa M Arbustini E Bosoni T Serio A Utility of Biochemical Markers in the Follow-Up of Heart Transplant Recipients. Transpl Proc (2003) 35(8):3075–8. 10.1016/j.transproceed.2003.10.044 14697983

[30] Patel PC Hill DA Ayers CR Lavingia B Kaiser P Dyer AK High-Sensitivity Cardiac Troponin I Assay to Screen for Acute Rejection in Patients with Heart Transplant. Circ Heart Fail (2014) 7(3):463–9. 10.1161/CIRCHEARTFAILURE.113.000697 24733367

[31] Muñoz-Esparza C Garrido IP Blanco R Casas T González-Cánovas C Pastor-Pérez F Usefulness of High Sensitivity Troponin T Assay in Detecting Acute Allograft Rejection after Heart Transplantation. Rev Esp Cardiol Engl (2011) 64(12):1109–13. 10.1016/j.rec.2011.06.017 21924812

[32] Dyer AK Barnes AP Fixler DE Shah TK Sutcliffe DL Hashim I Use of a Highly Sensitive Assay for Cardiac Troponin T and N-Terminal Pro-Brain Natriuretic Peptide to Diagnose Acute Rejection in Pediatric Cardiac Transplant Recipients. Am Heart J (2012) 163(4):595–600. 10.1016/j.ahj.2012.02.003 22520525

[33] Liu Z Perry LA Penny-Dimri JC Handscombe M Overmars I Plummer M Donor Cardiac Troponin for Prognosis of Adverse Outcomes in Cardiac Transplantation Recipients: A Systematic Review and Meta-Analysis. Transpl Direct (2021) 8(1):e1261. 10.1097/TXD.0000000000001261 PMC8670586 34912948

[34] Fitzsimons S Evans J Parameshwar J Pettit SJ. Utility of Troponin Assays for Exclusion of Acute Cellular Rejection after Heart Transplantation: A Systematic Review. J Heart Lung Transpl (2018) 37(5):631–8. 10.1016/j.healun.2017.12.008 29426716

[35] Hervás I Almenar L Pérez-Pastor JL Chirivella M Osa A Martínez-Dolz L Radioimmunometric Assay of B-Type Natriuretic Peptide (BNP) in Heart Transplantation: Correlation between BNP Determinations and Biopsy Grading of Rejection. Nucl Med Commun (2003) 24(8):925–31. 10.1097/01.mnm.0000084588.29433.2e 12869826

[36] Wu AH Johnson ML Aaronson KD Gordon D Dyke DBS Koelling TM. Brain Natriuretic Peptide Predicts Serious Cardiac Allograft Rejection Independent of Hemodynamic Measurements. J Heart Lung Transpl (2005) 24(1):52–7. 10.1016/j.healun.2003.10.012 15653379

[37] Rossano JW Denfield SW Kim JJ Price JF Jefferies JL Decker JA B-Type Natriuretic Peptide Is a Sensitive Screening Test for Acute Rejection in Pediatric Heart Transplant Patients. J Heart Lung Transpl (2008) 27(6):649–54. 10.1016/j.healun.2008.03.008 18503965

[38] Kittleson MM Skojec DV Wittstein IS Champion HC Judge DP Barouch LA The Change in B-Type Natriuretic Peptide Levels over Time Predicts Significant Rejection in Cardiac Transplant Recipients. J Heart Lung Transpl (2009) 28(7):704–9. 10.1016/j.healun.2009.04.019 19560699

[39] Damodaran A Dardas T Wu AH Dyke DBS Hummel SL Cowger JA Changes in Serial B-Type Natriuretic Peptide Level Independently Predict Cardiac Allograft Rejection. J Heart Lung Transpl (2012) 31(7):708–14. 10.1016/j.healun.2012.02.014 22502810

[40] Klingenberg R Koch A Gleissner C Schnabel PA Haass M Remppis A Determinants of B-Type Natriuretic Peptide Plasma Levels in the Chronic Phase after Heart Transplantation. Transpl Int (2005) 18(2):169–76. 10.1111/j.1432-2277.2004.00010.x 15691269

[41] Martínez-Dolz L Almenar L Hervás I Moro J Agüero J Sánchez-Lázaro I Prognostic Relationship between Two Serial Determinations of B-Type Natriuretic Peptide and Medium–Long-Term Events in Heart Transplantation. J Heart Lung Transpl (2008) 27(7):735–40. 10.1016/j.healun.2008.04.008 18582802

[42] Hammerer-Lercher A Mair J Antretter H Ruttmann E Poelzl G Laufer G B-Type Natriuretic Peptide as a Marker of Allograft Rejection after Heart Transplantation. J Heart Lung Transpl (2005) 24(9):1444–.e8. 10.1016/j.healun.2004.08.018 16143273

[43] Knecht KR Alexander ML Swearingen CJ Frazier EA. NTproBNP as a Marker of Rejection in Pediatric Heart Transplant Recipients: NTproBNP in Pediatric Heart Transplant. Pediatr Transpl (2012) 16(4):335–9. 10.1111/j.1399-3046.2012.01659.x 22429516

[44] Bader FM Rogers RK Kfoury AG Gilbert EM Horne BD Stehlik J Time-Dependent Changes in B-Type Natriuretic Peptide after Heart Transplantation: Correlation with Allograft Rejection and Function. Congest Heart Fail (2009) 15(2):63–7. 10.1111/j.1751-7133.2009.00055.x 19379451

[45] Lindblade CL Chun DS Darragh RK Caldwell RL Murphy DJ Schamberger MS. Value of Plasma B-Type Natriuretic Peptide as a Marker for Rejection in Pediatric Heart Transplant Recipients. Am J Cardiol (2005) 95(7):909–11. 10.1016/j.amjcard.2004.11.054 15781032

[46] Mehra MR Uber PA Potluri S Ventura HO Scott RL Park MH. Usefulness of an Elevated B-Type Natriuretic Peptide to Predict Allograft Failure, Cardiac Allograft Vasculopathy, and Survival after Heart Transplantation. Am J Cardiol (2004) 94(4):454–8. 10.1016/j.amjcard.2004.04.060 15325928

[47] Ambrosi P Oddoze C Ribéri A Arques S Portugal H Métras D Usefulness of N-Terminal–Pro-Brain Natriuretic Peptide Levels in Predicting Survival in Heart Transplant Recipients. Am J Cardiol (2004) 94(12):1585–7. 10.1016/j.amjcard.2004.08.049 15589026

[48] Almenar L Hervás I Martínez-Dolz L Rueda J Arnau MA Osa A The Value of Brain Natriuretic Peptide for the Diagnosis of Heart Transplant Rejection. Transpl Proc (2002) 34(1):174–5. 10.1016/s0041-1345(01)02716-6 11959236

[49] Arnau-Vives MA Almenar L Hervas I Osa A Martinez-Dolz L Rueda J Predictive Value of Brain Natriuretic Peptide in the Diagnosis of Heart Transplant Rejection. J Heart Lung Transpl (2004) 23(7):850–6. 10.1016/j.healun.2003.08.005 15261180

[50] O’Neill JO Mcrae AT Troughton RW Ng K Taylor DO Yamani MH Brain Natriuretic Peptide Levels Do Not Correlate with Acute Cellular Rejection in De Novo Orthotopic Heart Transplant Recipients. J Heart Lung Transpl (2005) 24(4):416–20. 10.1016/j.healun.2003.12.006 15797742

[51] Arora S Gullestad L Wergeland R Simonsen S Holm T Hognestad A Probrain Natriuretic Peptide and C-Reactive Protein as Markers of Acute Rejection, Allograft Vasculopathy, and Mortality in Heart Transplantation. Transplantation (2007) 83(10):1308–15. 10.1097/01.tp.0000263338.39555.21 17519779

[52] Jang MK Tunc I Berry GJ Marboe C Kong H Keller MB Donor-Derived Cell-Free DNA Accurately Detects Acute Rejection in Lung Transplant Patients, a Multicenter Cohort Study. J Heart Lung Transpl (2021) 40(8):822–30. 10.1016/j.healun.2021.04.009 PMC8319066 34130911

[53] De Vlaminck I Martin L Kertesz M Patel K Kowarsky M Strehl C Noninvasive Monitoring of Infection and Rejection after Lung Transplantation. Proc Natl Acad Sci (2015) 112(43):13336–41. 10.1073/pnas.1517494112 26460048 PMC4629384

[54] Rosenheck JP Ross DJ Botros M Wong A Sternberg J Chen YA Clinical Validation of a Plasma Donor-Derived Cell-Free DNA Assay to Detect Allograft Rejection and Injury in Lung Transplant. Transpl Direct (2022) 8(4):e1317. 10.1097/TXD.0000000000001317 PMC8963832 35372675

[55] Khush KK De Vlaminck I Luikart H Ross DJ Nicolls MR. Donor-derived, Cell-Free DNA Levels by Next-Generation Targeted Sequencing Are Elevated in Allograft Rejection after Lung Transplantation. ERJ Open Res (2021) 7(1):00462–2020. 10.1183/23120541.00462-2020 33532456 PMC7836440

[56] Keller MB Meda R Fu S Yu K Jang MK Charya A Comparison of Donor-Derived Cell-Free DNA between Single versus Double Lung Transplant Recipients. Am J Transpl (2022) 22(10):2451–7. 10.1111/ajt.17039 PMC9508279 35322546

[57] Agbor-Enoh S Jackson AM Tunc I Berry GJ Cochrane A Grimm D Late Manifestation of Alloantibody-Associated Injury and Clinical Pulmonary Antibody-Mediated Rejection: Evidence from Cell-Free DNA Analysis. J Heart Lung Transpl (2018) 37(7):925–32. 10.1016/j.healun.2018.01.1305 29500138

[58] Keller M Sun J Mutebi C Shah P Levine D Aryal S Donor-derived Cell-Free DNA as a Composite Marker of Acute Lung Allograft Dysfunction in Clinical Care. J Heart Lung Transpl (2022) 41(4):458–66. 10.1016/j.healun.2021.12.009 35063338

[59] Sayah D Weigt SS Ramsey A Ardehali A Golden J Ross DJ. Plasma Donor-Derived Cell-Free DNA Levels Are Increased during Acute Cellular Rejection after Lung Transplant: Pilot Data. Transpl Direct (2020) 6(10):e608. 10.1097/TXD.0000000000001063 PMC7515612 33062841

[60] Keller M Mutebi C Shah P Levine D Aryal S Iacono A Biological Variation of Donor-Derived Cell-Free DNA in Stable Lung Transplant Recipients. J Appl Lab Med (2022) 7(4):901–9. 10.1093/jalm/jfab171 35024828

[61] Agbor-Enoh S Wang Y Tunc I Jang MK Davis A De Vlaminck I Donor-Derived Cell-Free DNA Predicts Allograft Failure and Mortality after Lung Transplantation. EBioMedicine (2019) 40:541–53. 10.1016/j.ebiom.2018.12.029 30692045 PMC6412014

[62] Keller M Bush E Diamond JM Shah P Matthew J Brown AW Use of Donor-derived Cell-Free DNA as a Marker of Early Allograft Injury in Primary Graft Dysfunction (PGD) to Predict the Risk of Chronic Lung Allograft Dysfunction (CLAD). J Heart Lung Transpl (2021) 40(6):488–93. 10.1016/j.healun.2021.02.008 PMC8169618 33814284

[63] Bazemore K Permpalung N Mathew J Lemma M Haile B Avery R Elevated Cell-Free DNA in Respiratory Viral Infection and Associated Lung Allograft Dysfunction. Am J Transpl (2022) 22(11):2560–70. 10.1111/ajt.17125 35729715

[64] Shah P Agbor-Enoh S Bagchi P deFilippi CR Mercado A Diao G Circulating microRNAs in Cellular and Antibody-Mediated Heart Transplant Rejection. J Heart Lung Transpl (2022) 41:1401–13. 10.1016/j.healun.2022.06.019 PMC9529890 35872109

[65] Sukma Dewi I Celik S Karlsson A Hollander Z Lam K McManus JW Exosomal miR-142-3p Is Increased during Cardiac Allograft Rejection and Augments Vascular Permeability through Down-Regulation of Endothelial RAB11FIP2 Expression. Cardiovasc Res (2017) 113:440–52. 10.1093/cvr/cvw244 28073833

[66] Duong Van Huyen JP Tible M Gay A Guillemain R Aubert O Varnous S MicroRNAs as Non-Invasive Biomarkers of Heart Transplant Rejection. Eur Heart J (2014) 35:3194–202. 10.1093/eurheartj/ehu346 25176944

[67] Castellani C Burrello J Fedrigo M Burrello A Bolis S Di Silvestre D Circulating Extracellular Vesicles as Non-Invasive Biomarker of Rejection in Heart Transplant. J Heart Lung Transpl (2020) 39:1136–48. 10.1016/j.healun.2020.06.011 32665078

[68] Constanso-Conde I Hermida-Prieto M Barge-Caballero E Núñez L Pombo-Otero J Suárez-Fuentetaja N Circulating miR-181a-5p as a New Biomarker for Acute Cellular Rejection in Heart Transplantation. J Heart Lung Transpl (2020) 39:1100–8. 10.1016/j.healun.2020.05.018 32654912

[69] Kennel PJ Saha A Maldonado DA Givens R Brunjes DL Castillero E Serum Exosomal Protein Profiling for the Non-Invasive Detection of Cardiac Allograft Rejection. J Heart Lung Transpl (2018) 37:409–17. 10.1016/j.healun.2017.07.012 28789823

[70] Habertheuer A Korutla L Rostami S Reddy S Lal P Naji A Donor Tissue-Specific Exosome Profiling Enables Noninvasive Monitoring of Acute Rejection in Mouse Allogeneic Heart Transplantation. J Thorac Cardiovasc Surg (2018) 155:2479–89. 10.1016/j.jtcvs.2017.12.125 29499866

[71] Peyster EG Arabyarmohammadi S Janowczyk A Azarianpour-Esfahani S Sekulic M Cassol C An Automated Computational Image Analysis Pipeline for Histological Grading of Cardiac Allograft Rejection. Eur Heart J (2021) 42:2356–69. 10.1093/eurheartj/ehab241 33982079 PMC8216729

[72] Peyster EG Wang C Ishola F Remeniuk B Hoyt C Feldman MD *In Situ* Immune Profiling of Heart Transplant Biopsies Improves Diagnostic Accuracy and Rejection Risk Stratification. JACC Basic Transl Sci (2020) 5:328–40. 10.1016/j.jacbts.2020.01.015 32368693 PMC7188920

[73] Peyster EG Janowczyk A Swamidoss A Kethireddy S Feldman MD Margulies KB. Computational Analysis of Routine Biopsies Improves Diagnosis and Prediction of Cardiac Allograft Vasculopathy. Circulation (2022) 145:1563–77. 10.1161/circulationaha.121.058459 35405081 PMC9133227

[74] Halloran PF Madill-Thomsen KS. The Molecular Microscope Diagnostic System: Assessment of Rejection and Injury in Heart Transplant Biopsies. Transplantation (2023) 107:27–44. 10.1097/tp.0000000000004323 36508644

[75] Coutance G Desiré E Duong Van Huyen JP. A Review of Biomarkers of Cardiac Allograft Rejection: Toward an Integrated Diagnosis of Rejection. Biomolecules (2022) 12:1135. 10.3390/biom12081135 36009029 PMC9405997

[76] Halloran PF Potena L Van Huyen JD Bruneval P Leone O Kim DH Building a Tissue-Based Molecular Diagnostic System in Heart Transplant Rejection: The Heart Molecular Microscope Diagnostic (MMDx) System. J Heart Lung Transpl (2017) 36:1192–200. 10.1016/j.healun.2017.05.029 28662985

[77] Alam A Van Zyl J Paul Milligan G Michelle McKean S Patel R Anne Hall S. Evolving the Surveillance and Workup of Heart Transplant Rejection: A Real-World Analysis of the Molecular Microscope Diagnostic System. Am J Transpl (2022) 22:2443–50. 10.1111/ajt.17087 35514138

[78] Dolan RS Rahsepar AA Blaisdell J Suwa K Ghafourian K Wilcox JE Multiparametric Cardiac Magnetic Resonance Imaging Can Detect Acute Cardiac Allograft Rejection after Heart Transplantation. JACC Cardiovasc Imaging (2019) 12:1632–41. 10.1016/j.jcmg.2019.01.026 30878427 PMC6995349

[79] Imran M Wang L McCrohon J Yu C Holloway C Otton J Native T(1) Mapping in the Diagnosis of Cardiac Allograft Rejection: A Prospective Histologically Validated Study. JACC Cardiovasc Imaging (2019) 12:1618–28. 10.1016/j.jcmg.2018.10.027 30660547

[80] Miller CA Naish JH Shaw SM Yonan N Williams SG Clark D Multiparametric Cardiovascular Magnetic Resonance Surveillance of Acute Cardiac Allograft Rejection and Characterisation of Transplantation-Associated Myocardial Injury: A Pilot Study. J Cardiovasc Magn Reson (2014) 16:52. 10.1186/s12968-014-0052-6 25160654 PMC4121512

[81] Anthony C Imran M Pouliopoulos J Emmanuel S Iliff J Liu Z Cardiovascular Magnetic Resonance for Rejection Surveillance after Cardiac Transplantation. Circulation (2022) 145:1811–24. 10.1161/CIRCULATIONAHA.121.057006 35621277

